# Structure of a model lipid membrane oxidized by human 15-lipoxygenase-2

**DOI:** 10.1016/j.bbrc.2024.150533

**Published:** 2024-08-08

**Authors:** Jamil Nemri, Cosme Morales, Nathaniel C. Gilbert, Jaroslaw Majewski, Marcia E. Newcomer, Crystal M. Vander Zanden

**Affiliations:** a Department of Chemistry and Biochemistry, University of Colorado Colorado Springs, 1420 Austin Bluffs Pwky, Colorado Springs, CO, 80918, USA; b Department of Biological Sciences, Louisiana State University, 202 Life Sciences Building, Baton Rouge, LA, 70803, USA; c Division of Molecular and Cellular Biosciences, National Science Foundation, Alexandria, VA, USA; d Theoretical Biology and Biophysics at Los Alamos National Laboratory, Los Alamos National Laboratory, Los Alamos, NM, 87545, USA; e Department of Chemical and Biological Engineering and Center for Biomedical Engineering, University of New Mexico, Albuquerque, NM, 87131, USA

**Keywords:** Lipoxygenase, Oxidation, Arachidonic acid, X-ray reflectivity, Membranes, 15-Lipoxygenase-2

## Abstract

Enzyme-mediated lipid oxidation is an important regulatory event in cell signaling, with oxidized lipids being potent signaling molecules that can illicit dramatic changes in cell behavior. For example, peroxidation of an arachidonoyl poly-unsaturated fatty acid by the human enzyme 15-lipoxygenase-2 (15-LOX-2) has been associated with formation of atherosclerotic plaques. Previous work on synthetically oxidized membranes has shown that oxidized lipid tails will change their conformation to facilitate interactions between the peroxide group and the lipid headgroups. However, this phenomenon has not been directly observed for a lipid membrane that has undergone enzyme-catalyzed oxidation. In this study, we report on the structure of a model lipid membrane before and after oxidation by 15-LOX-2. A model lipid membrane monolayer at the air-liquid interface was constructed from 1-stearoyl-2-arachidonoyl-*sn*-glycero-3-phosphocholine (SAPC) in a Langmuir trough, and X-ray reflectivity measurements were conducted to determine the electron density profile of the system. Exposure to 15-LOX-2 caused a dramatic change in the SAPC structure, namely a blurred distinction between the lipid tail/head layers and shortening of the average lipid tail length by ~3 Å. The electron density profile of the oxidized SAPC monolayer is similar to that of a synthetically oxidized substrate mimic. Overall, this reported observation of an enzymatically-oxidized membrane structure in situ is helping to bridge a gap in the literature between structural studies on synthetically oxidized membranes and cellular studies aiming to understand physiological responses.

## 1. Introduction

**Fig. 1.**
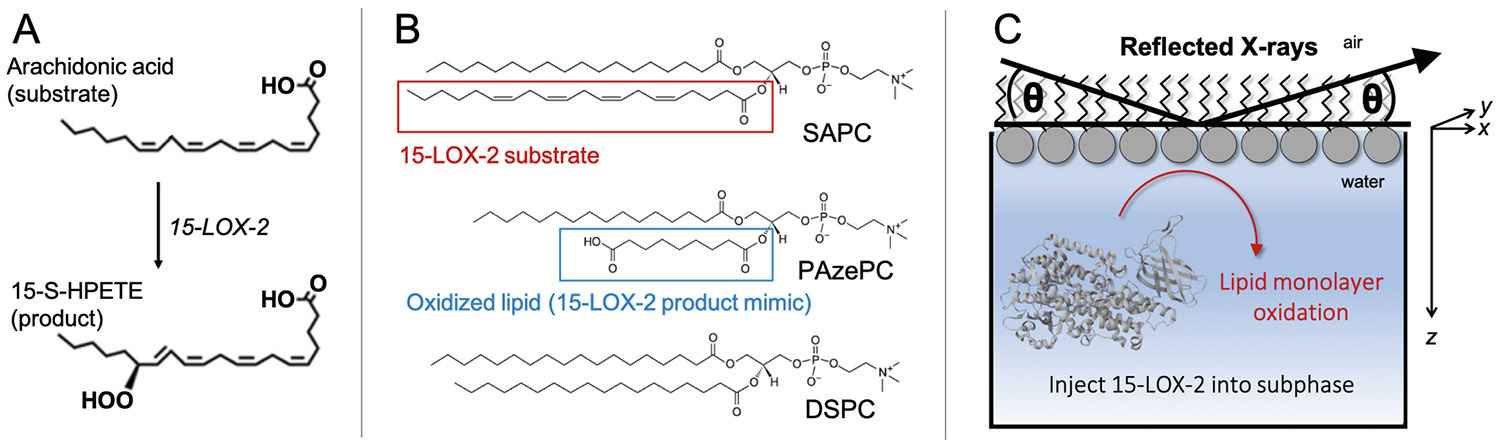
Schematic of experimental design and lipid structures. (A) Human 15-lipoxygenase-2 (15-LOX-2) catalyzes peroxidation of an arachidonic acid substrate to form 15-S-HPETE product. (B) Structure of each lipid used. (C) Liquid surface X-ray reflectivity measurements were performed on a lipid monolayer formed at the air/water interface in a Langmuir trough. 15-LOX-2 [19] was injected into the water subphase beneath the monolayer and allowed to interact with the membrane.

Enzyme-mediated lipid oxidation has emerged as a developing topic important for understanding a variety of diseases including diabetes, arthritis, and atherosclerosis [1]. Lipid oxidation is a critical phenomenon in cell signaling, with enzymatically oxidized poly-unsaturated fatty acids serving as potent signaling molecules that elicit dramatic changes in cell behavior [2,3]. One example are the lipids oxidized by the peripheral membrane protein 15-lipoxygenase-2 (15-LOX-2), an enzyme expressed in macrophages and localized to the cell membrane via calcium ion signaling. Through iron-mediated catalysis, 15-LOX-2 binds an arachidonate substrate and generates a 15-hydroperoxyeicosatetraenoic acid (15-HPETE) product (Fig. 1A) [4]. The resulting oxidized lipids can be packaged into low-density lipoproteins and taken up by macrophages, causing transformation to foam cells, further inflammation, and finally formation of atherosclerotic plaques [5-7]. Importantly, 15-LOX-2 is able to bind the cell membrane and initiate catalysis without aid from accessory proteins, unlike some other proteins in the lipoxygenase family [8]. In particular, cytosolic phospholipase A2 usually liberates the esterified arachidonyl from the membrane to generate free arachidonic acid, which is the common substrate for most lipoxygenases. 15-LOX-2 acts on a variety of arachidonoyl phospholipid substrates with different headgroups, and its activity has been confirmed using a variety of methods including cell assays, mass spectrometry, and activity assays using lipid nanodiscs [9,10].

Given the relevance of these oxidized lipids in cellular functions, it is important to understand how they are capable of modifying membrane structure to elicit recognition by proteins. Research on this topic has focused on synthetically oxidized lipids generated through chemical/physical processes such as oxidizers or UV light. Through neutron and X-ray scattering, molecular dynamics simulations, and microscopy, work on these artificial membranes has illustrated that oxidized lipids cause concentration-dependent bilayer thinning, increased area per lipid, membrane permeability, and membrane deformation [11-15]. Analysis of differing peroxidation locations shows that modifications towards the omega end of the acyl tail have larger impacts than oxidation closer to the carbonyl [16]. Further, oxidized lipids are expected to cluster together into domains with higher microviscosity, driven by increased hydrogen bonding interactions between peroxidized lipid tails [17]. Altogether, structural work on synthetically oxidized membranes support the “lipid whisker” model, where oxidation sites on acyl tails are expected to float towards the membrane surface to increase hydrophilic interactions with the lipid headgroups [18]. This modified membrane structure exposes the oxidized “whisker” and remaining portion of the acyl tail to the membrane surface, allowing for convenient recognition by oxidation-sensing proteins. Despite the reasonably good characterization of these synthetically oxidized membranes, to our knowledge, there have been no reports directly characterizing an enzyme-oxidized membrane structure.

We report the structure of a membrane that has undergone oxidation in-situ by the human 15-LOX-2 enzyme. These experiments were designed using liquid surface X-ray reflectivity (XR) measurements from a lipid monolayer deposited at an air/water interface in a Langmuir trough before and after exposure to the 15-LOX-2 enzyme (Fig. 1). Overall, our findings indicate that 15-LOX-2 was able to oxidize the arachidonoyl substrate in this context of a lipid monolayer composed of 1-stearoyl-2-arachidonoyl-*sn*-glycero-3-phosphocholine (SAPC). Lipid oxidation caused a major membrane structure change, consistent with the peroxide-modified C15 adjusting its position to interact among the lipid headgroups. These results were compared to XR measurements from a commercially-available oxidized lipid used as a substrate mimic, demonstrating reasonably good agreement between the membrane structures.

## 2. Materials and methods

### Materials:

1,2-distearoyl-*sn*-glycero-3-phosphocholine (DSPC), 1-stearoyl-2-arachidonoyl-*sn*-glycero-3-phosphocholine (SAPC), and 1-palmitoyl-2-azelaoyl-*sn*-glycero-3-phosphocholine (PAzePC) were obtained from Avanti Polar Lipids (Alabaster, AL). For Langmuir trough monolayer preparation, lipids were dissolved in 8:2 chloroform:methanol at 0.2 mg/mL. Wild type 15-LOX-2 protein was expressed and purified as previously described [19].

### X-ray Reflectivity Data Collection:

X-ray reflectivity (XR) data was collected at Sector 15 NSF’s ChemMatCARS at the Advanced Photon Source at Argonne National Labs. The room temperature (23.5 ± 0.5 °C) subphase was composed of degassed buffer containing 150 mM NaCl, 20 mM Tris pH7.4, and 0.5 mM EDTA. Surface pressure in the 20 mL Langmuir trough (6.5 × 6.5 cm^2^) was measured with a KSV Instruments Wilhelmy plate balance. Lipids were spread to a 25 mN/m surface pressure, and solvent was allowed to evaporate for 10min before XR data collection on the pure membrane. 15-LOX-2 was then injected to a final concentration of 3 μM in the aqueous subphase, and the protein was incubated with the membrane for 4h prior to data collection.

The Langmuir trough was placed within a sealed canister, and the system was flushed with helium gas to keep the O_2_ content <2 %. This was maintained during X-ray exposure to reduce oxidative beam damage to the monolayer and background scattering. The X-ray wavelength was 1.24 Å, and the incoming X-ray beam contacted the liquid surface with a footprint sized around ~1x3-10 mm^2^. The trough was moved 1 mm after each scan, so data was continuously being collected on a new area of membrane to reduce beam damage. A germanium monochromator crystal was used to change the angle of incidence on the sample, collecting data over the range of 0.016 *< q*_z_ < 0.7 Å^−1^. Reflectivity measurements were captured with a Dectris PILATUS 100K detector, then normalized to incident beam flux and background subtracted. Python software was used to integrate the images [20]. Data were normalized to the Fresnel reflectivity (*R_F_*), which is the reflectivity expected from an infinitely sharp air-water interface. The error bars shown in the datasets represent one standard deviation error.

### 2.1. X-ray reflectivity theory and data analysis

The theory of liquid surface XR has been previously described [21-24]. To briefly summarize, the XR measurement is the intensity of the reflected beam normalized to the incident beam, in specular geometry. Reflectivity is collected as a function of the vertical momentum transfer vector (*q*_*z*_). *q*_*z*_ is related to the incident angle (θ) and the X-ray wavelength (*λ*) through the expression *q*_z_=(4π/λ)*sin*θ*. Through analysis using the recursive Parratt formalism [25], XR can be used to determine the electron density profile normal to the air/water interface, *ρ(z)*, of materials deposited as thin films on the surface of a Langmuir trough. The intensity of reflected X-rays can be analyzed to produce a model of *ρ(z)* as a function of depth that represents the laterally averaged structure of molecules illuminated by the beam.

Two complementary data-fitting approaches were applied. The first approach used a model-free fitting procedure based on cubic B-splines [26], where constrained nonlinear least-squares methods were used to determine the coefficients in the B-spline series. Thousands of models were produced, and the best model was selected from the smoothest profile with the lowest χ^2^ [27]. For each dataset, a family of fit models are presented that all fall within $\chi^{2}\leq \chi_{\text{min}}^{2}+20\%$, represented by the dashed lines (ribbons) shown bounding the best fit model in *R/R_F_* data plots. Corresponding uncertainties are also represented by dashed lines bounding the ρ/ρ_water_ profiles. The advantage of this fitting method is the high confidence in the resulting model and the reduced impact of fitting bias.

A second analysis method was used, which relied on iterative refinement of a single model. Using the program Motofit [28], the model was built with unique parameters describing the lipid tails, headgroups, and subphase, which altogether constructed a unified profile that assumed the film was composed of discreet layers of electron density. For each layer, floating parameters were fitted for the electron density, length, and interfacial roughness approximated by error functions. The best model was chosen based on having the lowest χ^2^ values while maintaining realistic parameter values. In some cases, a parameter was fixed to reduce the number of variables and improve uncertainty in the remaining floating parameters. The errors reported for each fitted parameter were obtained by measuring the concavity of χ^2^ using a finite difference approach with a covariance matrix. However, it should be noted that some parameter errors may be underestimated due the number of floating parameters and their interdependence. Altogether, the combination of the two fitting methods provides robust analysis of the data, especially when considered together.

## 3. Results

We report here the structure of an SAPC membrane before and after oxidation of the arachidonoyl substrate by the human 15-LOX-2 enzyme. Importantly, these measurements describe *in-situ* membrane structure changes as a result of direct lipid oxidation by an enzyme. Although this is a simplified model system, the design was intended to amplify the modified SAPC structure changes so oxidation effects could be clearly observed.

To determine membrane structure, liquid surface XR measurements were performed from a lipid monolayer formed at the air/water interface in a Langmuir trough. This method was chosen because it produces a sensitive measurement of the film, which can distinguish small differences in electron density, including those of the lipid hydrocarbon tails and phosphate headgroups. Electron dense material at the water surface produces changes in the measured reflectivity (*R*) compared to the Fresnel reflectivity from an ideal interface (*R*_*F*_). A profile of electron density normalized to solvent (ρ/ρ_water_) as a function of depth into the membrane (along the z-axis) can be used to deduce membrane structure and identify changes caused by enzyme-mediated oxidation. ρ/ρ_water_ profiles were determined using two complementary R/R_F_ fitting methods (model-independent and -dependent), which together produce a robust approach that yields parameterized values for each chemical moiety while also reducing the impact of fitting bias.

**Fig. 2.**
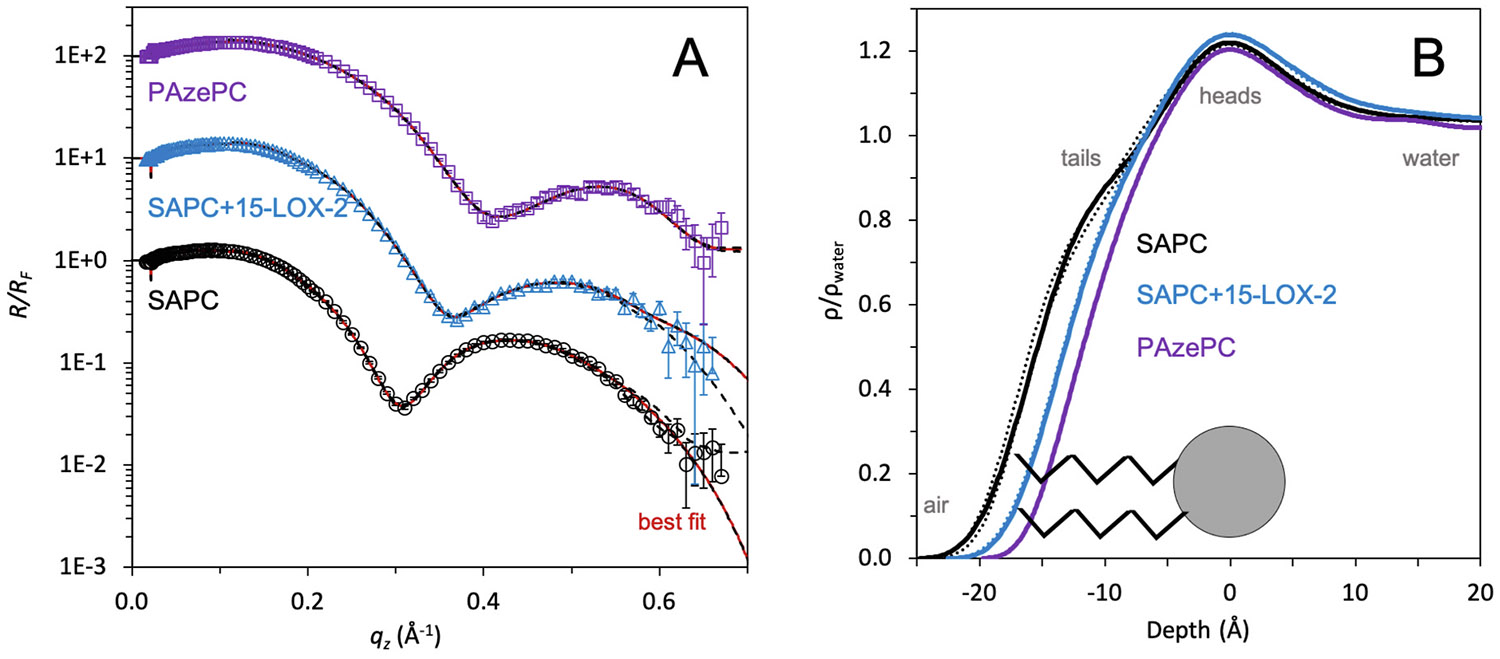
15-LOX-2 oxidizes a lipid monolayer containing an arachidonoyl substrate. (A) X-ray reflectivity data and model-independent fitting for lipid monolayers composed of SAPC, SAPC+15-LOX-2, and PAzePC. Normalized reflectivities (*R/R*_*F*_) are plotted as a function of the vertical momentum transfer vector (*q*_*z*_). Measured reflectivities are represented as points with experimental error. The plot shows the best-fit model (red line) and the outer bound of all models within 20 % of the lowest χ^2^ as black dashed lines on either side of the best-fit line. The bounding models lie on top or near the best-fit model, indicating high confidence in the fit. Reflectivities are shown with a vertical offset for clarity. (B) Normalized electron density profiles (*ρ*/*ρ*_water_) from reflectivity fitting are plotted as a function of depth along *z*, normal to the air/water interface, where zero is defined as the center of the lipid headgroup. The lowest χ^2^ model is shown as a solid line. All models within 20 % of the lowest χ^2^ are bounded within dashed lines, although these are nearly overlaying the best-fit model. A cartoon lipid is overlaid on the electron density profile.

Data were first collected on the pure SAPC monolayer. The results of model-independent fitting to the *R/R*_*F*_ data show good agreement between the fit and the data, with the upper and lower-bounding ribbons nearly overlaying the best fit line (Fig. 2A). Model-dependent fit profiles (Fig. S1) and parameters (Table 1) also suggest that the resulting *ρ/ρ*_*water*_ profile can be achieved through reasonable parameters for the lipid tails and heads. The good fit to the data, as well as the good match between both fitting methods, indicates high confidence in the resulting electron density profiles. The resulting normalized electron density profile is plotted as a function of depth along the *z* dimension (normal to the air/water interface) (Fig. 2B). Bounding ribbons are also overlaid onto the *ρ/ρ*_*water*_ profiles, but they are nearly overlapping the best fit model.

The SAPC *ρ/ρ_water_* profile revealed two distinguishable layers of electron density representing the lipid tails and headgroups (Fig. 2B). The lipid tails were fitted to a length of 11.8 Å, and the headgroups had a length of 6.3 Å (Table 1). Very large roughness values were required to parameterize the profile, which is likely caused by the high degree of disorder in the arachidonoyl tail with four unsaturated bonds and possible related membrane instability. Grazing incidence X-ray diffraction (GIXD) data was also collected on this sample (not shown), which revealed no measurable diffraction peaks above background scattering. This supports the understanding that the pure SAPC membrane at a surface pressure of 25 mN/m is in a relatively disordered conformation, with no measurable ordered packing between lipid tails.

After data was collected on the pure SAPC monolayer, 15-LOX-2 protein was injected into the aqueous subphase below the membrane and the protein was allowed to interact with the membrane for 4h. Then the XR data was recollected on the same membrane, allowing observation of any membrane changes that resulted from protein activity. After 15-LOX-2 activity, the membrane structure was distinctively changed (Fig. 2B). There was a significant reduction in the lipid tail length by ~3 Å, shortening the lipid tails to 8.6 Å (Table 1). The measured XR data represents the average lipid structure for billions of molecules that lie within the footprint of the coherent area of the X-ray beam, so the lipid tail length represents the average of both the unsaturated stearoyl tail and the potentially oxidized arachidonoyl tail in SAPC after 15-LOX-2 activity. A reasonable explanation for the shortened tail length is the oxidized portion of the lipid is drawn to interact with the hydrophilic headgroups, bending the arachidonoyl tail, and therefore shortening the observed average lipid tail length.

As a positive control, XR data was also collected from a pure PAzePC membrane, which was intended as a mimic for the oxidized SAPC product resulting from 15-LOX-2 activity. In general, the measured structures of the oxidized SAPC and PAzePC membranes are similar. A difference was observed in the length of the PAzePC membrane, being ~1 Å shorter than the oxidized SAPC. This shorter tail is not surprising, especially because the palmitoyl tail is two carbons shorter than the stearoyl tail. With both of these lipid tails being fully saturated and unoxidized, they are expected to be in a somewhat extended conformation and relatively unimpacted by their neighboring oxidized tail.

Interestingly, the 15-LOX-2 enzyme did not remain stably bound to the membrane. If the enzyme had remained bound, an additional layer of electron density would be observed beneath the lipid headgroups extending out towards the water subphase. 15-LOX-2 is a peripheral membrane-binding protein, so it is not expected to penetrate deeply into the monolayer. However, this lack of stable 15-LOX-2 binding is not altogether surprising because these experiments were performed in the absence of Ca^2+^ ions, which are known to accelerate 15-LOX-2 activity and encourage binding to the membrane [9]. Instead it appears that in this context 15-LOX-2 is transiently binding the membrane, performing catalysis, and departing.

**Fig. 3.**
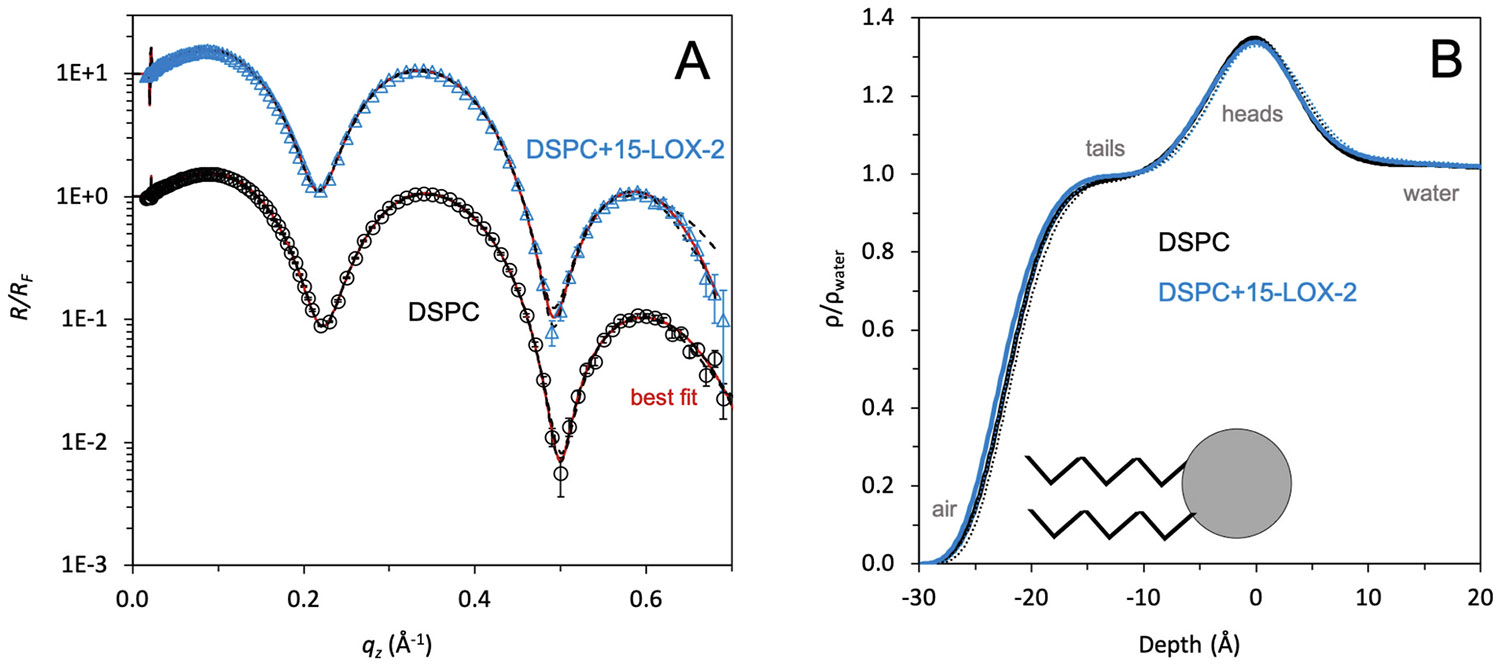
15-LOX-2 does not modify a membrane lacking substrate. (A) XR data and model-independent fitting for DSPC and DSPC+15-LOX-2, and (B) Normalized electron density profiles (*ρ*/*ρ*_water_) from this data, presented similarly to those in Fig. 2.

Finally, as a negative control, we measured the structure of a pure DSPC membrane alone and after exposure to 15-LOX-2 (Fig. 3). The pure DSPC membrane exhibited two distinctly separated regions for the lipid tails and heads. The lipid tails were parameterized to a length of ~18 Å and the heads had a length of ~8 Å. GIXD data collected from the DSPC membrane showed a clear diffraction peak indicating the acyl tails were coherently packed in a distorted hexagonal configuration with a unit cell length of 5.06 Å (data not shown). This longer tail length and coherent lipid packing was expected from a membrane containing fully saturated 18-carbon tails. The subphase roughness value, indicating the interfacial roughness between the lipid heads and the subphase, for the pure DSPC membrane was also much smaller than the pure SAPC membrane subphase roughness. This suggests the pure DSPC membrane is well-stratified and does not include major structural features or membrane instability which deviate from the monolayer structure. This is consistent with the membrane containing well-packed lipid tails in an ordered conformation.

After 4h of 15-LOX-2 incubation with the pure DSPC membrane, no major membrane structure changes were observed (Fig. 3, Table 1). This suggests that 15-LOX-2 was not engaging in non-specific membrane interactions.

## 4. Discussion and conclusion

In conclusion, we have observed membrane structure changes as a result of arachidonoyl substrate oxidation by 15-LOX-2. Membrane oxidation caused a major structural rearrangement, namely a reduced distinction between the lipid tail and headgroup electron density layers. The observed lipid tail length was effectively shortened by ~30 %, which is likely the result of rearrangement of the oxidized tail to form a bent structure with the peroxide oriented towards the lipid headgroups. Another factor is the measured ρ/ρ_water_ for the oxidized SAPC headgroup matches our calculated value given the number of electrons expected after peroxidation. In a hypothetical calculation excluding the peroxidation, the ρ/ρ_water_ is reduced from 1.48 to 1.40, suggesting the experimentally measured values confirm the presence of increased electrons added by peroxidation (Table S1). Furthermore, the measured structure of the enzyme-oxidized SAPC membrane is generally consistent with measurements from a synthetically oxidized substrate mimic.

Interestingly, the oxidation event occurred without direct observation of stably-bound 15-LOX-2 protein. This suggests a possible hopping mechanism for 15-LOX-2 in this context, where the protein has the ability to transiently bind the membrane, perform catalysis, and then depart, leaving an oxidized lipid embedded in the membrane. Importantly, these experiments were performed in the absence of Ca^2+^ ions, which are known to drive 15-LOX-2 binding to the membrane. It has previously been observed that 15-LOX-2 is able to bind a membrane and perform catalysis in the absence of Ca^2+^, although the enzyme activity is slower [9]. Our results are consistent with this finding. Of note, we did attempt experiments in the presence of calcium ions, but technical challenges arose that caused sample damage in the X-ray data collection environment, which prevented collection of good-quality data when Ca^2+^ was present.

Altogether, this work supports the “lipid whisker model” of lipid oxidation, where oxidized lipid tails are expected to rise to the membrane surface, providing easy access for recognition by downstream cell signaling proteins [18]. This finding is significant because it helps bridge a gap between in vitro characterization of synthetically oxidized membranes and in vivo studies determining the impact of 15-LOX-2 activity on cell behavior. In the future, further characterization of lipid oxidation and the related enzymes will lead to a better understanding of how to control cellular changes related to lipid oxidation events.

## Supplementary Material

supplement

## Figures and Tables

**Table 1:** Parameters obtained from model-dependent fitting of XR data.

	Layer1-Tails			Layer2-Headgroups			Subphase	
			
	Thickness (Å)	*ρ*/*ρ*_water_	Roughness (Å)	Thickness (Å)	*ρ*/*ρ*_water_	Roughness (Å)	Roughness (Å)	χ^2^
SAPC	11.8 ± 0.2	0.96 ± 0.01	3.66^a^	6.3 ± 1.3	1.35 ± 0.07	2.7 ± 0.2	4.9 ± 0.5	4.5
SAPC+15-LOX-2	8.64 ± 0.02	0.86 ± 0.01	3.11^a^	6.5 ± 1.4	1.48 ± 0.09	3.6 ± 0.2	7.1 ± 0.6	6.8
PAzePC	7.8 ± 0.3	0.86 ± 0.02	3.05^a^	6 ± 2	1.4 ± 0.2	3.4 ± 0.5	5.1 ± 0.8	5.2
								
DSPC	18.0 ± 0.2	1.00 ± 0.01	3.06 ± 0.02	7.7 ± 0.3	1.43 ± 0.02	3.6 ± 0.2	2.5 ± 0.1	2.4
DSPC+15-LOX-2	18.7 ± 0.2	1.03 ± 0.01	3.33 ± 0.02	7.5 ± 0.2	1.41 ± 0.02	2.5 ± 0.1	2.6 ± 0.1	3.1

a Parameter was fixed during fitting.

## Appendix A. Supplementary data

Supplementary data to this article can be found online at https://doi.org/10.1016/j.bbrc.2024.150533.

## Abbreviations

DSPC: 1,2-distearoyl-sn-glycero-3-phosphocholine
SAPC: 1-stearoyl-2-arachidonoyl-sn-glycero-3-phosphocholine
PAzePC: 1-palmitoyl-2-azelaoyl-sn-glycero-3-phosphocholine
15-LOX-2: wild type human 15-lipoxygenase-2
XR: X-ray reflectivity
GIXD: grazing incidence X-ray diffraction
