# Supplementary material for: Structure of a model lipid membrane oxidized by human 15-lipoxygenase-2
Source: Biochem Biophys Res Commun. Author manuscript; Available in PMC 2026 Jan 8. (PMC12782027; doi:10.1016/j.bbrc.2024.150533)

**SUPPLEMENTARY MATERIAL FOR**

Structure of a Model Lipid Membrane Oxidized by Human 15-Lipoxygenase-2

Jamil Nemri,^a^ Cosme Morales,^a^ Nathaniel C. Gilbert,^b^ Jaroslaw Majewski,^cde^ Marcia E. Newcomer,^b^ Crystal M. Vander Zanden^a*^

a. Department of Chemistry and Biochemistry, University of Colorado Colorado Springs, 1420 Austin Bluffs Pwky, Colorado Springs, CO 80918, USA. ([jnemri@uccs.edu](mailto:jnemri@uccs.edu); [cmorale4@uccs.edu](mailto:cmorale4@uccs.edu); [cvanderz@uccs.edu](mailto:cvanderz@uccs.edu))

b. Department of Biological Sciences, Louisiana State University, 202 Life Sciences Building, Baton Rouge, Louisiana 70803, USA. ([ngilbert@lsu.edu](mailto:ngilbert@lsu.edu); [newcomer@lsu.edu](mailto:newcomer@lsu.edu))

c. Division of Molecular and Cellular Biosciences, National Science Foundation, Alexandria, Virginia, USA. ([jmajewsk@nsf.gov](mailto:jmajewsk@nsf.gov))

d. Theoretical Biology and Biophysics at Los Alamos National Laboratory, Los Alamos National Laboratory, Los Alamos, New Mexico 87545, USA.

e. Department of Chemical and Biological Engineering and Center for Biomedical Engineering, University of New Mexico, Albuquerque, NM 87131, USA.

***Corresponding author:** Crystal Vander Zanden ([cvanderz@uccs.edu](mailto:cvanderz@uccs.edu))

**Supplementary Figures:**

**Figure S1:** Model-dependent fitting of XR data for SAPC, SAPC+15-LOX-2, and PAzePC.

**Figure S2:** Model-dependent fitting of XR data for DSPC and DSPC+15-LOX-2.

**Supplementary Table:**

**Table S1:** Number of atoms expected in each layer and resulting calculated ρ/ρ_water_ values

**Supplementary Figures:**

**
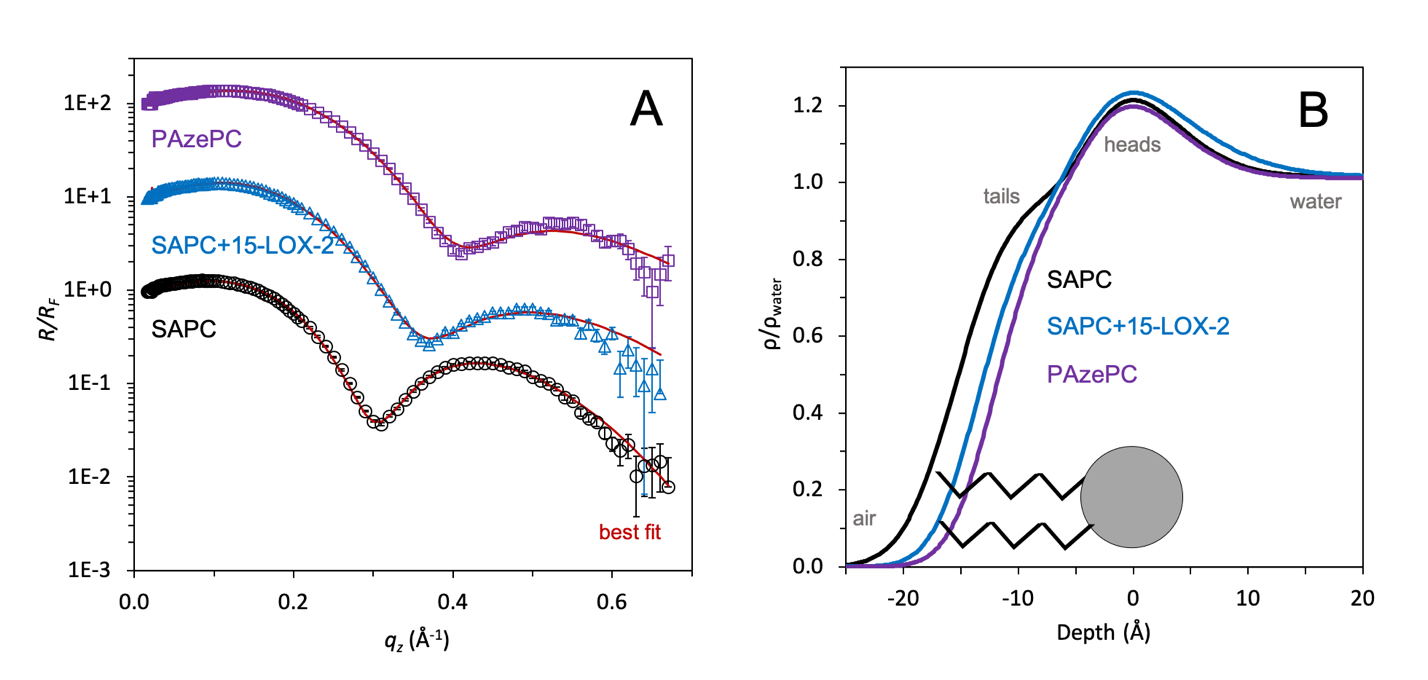
Figure S1:** Model-dependent fitting of X-ray reflectivity data for SAPC, SAPC+15-LOX-2, and PAzePC. **(A)** Normalized reflectivities (*R/R_F_*) are plotted as a function of the vertical momentum transfer vector (*q_z_*). Measured reflectivities are represented as points with experimental error. The plot shows the best-fit model overlaid as a red line. Reflectivities are shown with a vertical offset for clarity. **(B)** Normalized electron density profiles (*ρ*/*ρ*_water_) from reflectivity fitting are plotted as a function of depth along *z*, normal to the air/water interface, where zero is defined as the center of the lipid head group. A cartoon lipid is overlaid on the electron density profile.


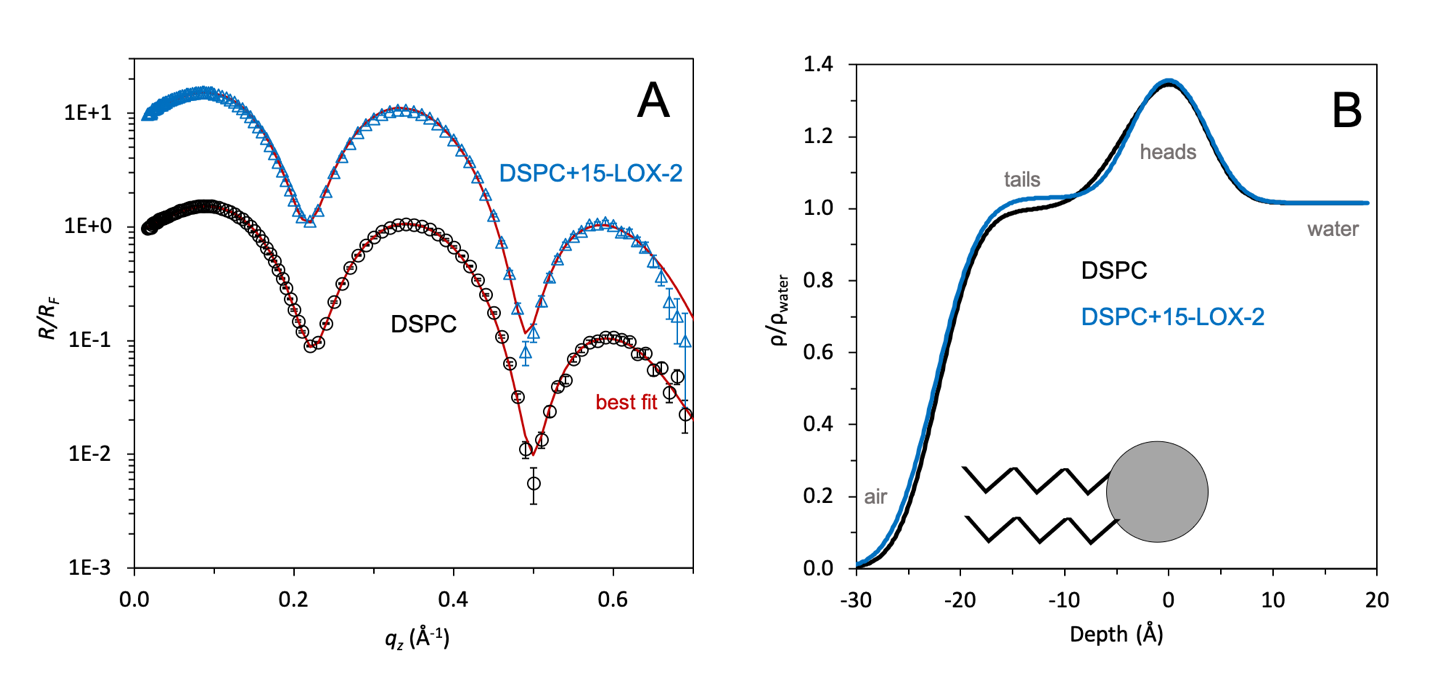


**Figure S2:** Model-dependent fitting of X-ray reflectivity data for DSPC and DSPC+15-LOX-2. **(A)** Normalized reflectivities (*R/R_F_*) are plotted as a function of the vertical momentum transfer vector (*q_z_*). Measured reflectivities are represented as points with experimental error. The plot shows the best-fit model overlaid as a red line. Reflectivities are shown with a vertical offset for clarity. **(B)** Normalized electron density profiles (*ρ*/*ρ*_water_) from reflectivity fitting are plotted as a function of depth along *z*, normal to the air/water interface, where zero is defined as the center of the lipid head group. A cartoon lipid is overlaid on the electron density profile.

**Supplementary Table:**

**Table S1:** Number of atoms expected in each layer and resulting calculated ρ/ρ_water_ values. A visual representation of the lipids is shown beneath the table.

| **Number of Atoms in Each Layer** | | | | | |
| --- | --- | --- | --- | --- | --- |
|  |  |  |  |  |  |
|  | SAPC Tails | SAPC Heads | Oxidized SAPC Tails | Oxidized SAPC Heads | Hypothetical: SAPC Heads without peroxidation |
| **Phosphatidylcholine headgroup** |  |  |  |  |  |
| C (6 e-) | - | 10 | - | 10 | 10 |
| N (7 e-) | - | 1 | - | 1 | 1 |
| O (8 e-) | - | 8 | - | 8 | 8 |
| H (1 e-) | - | 18 | - | 18 | 18 |
| P (15 e-) | - | 1 | - | 1 | 1 |
|  |  |  |  |  |  |
| **Stearoyl tail** |  |  |  |  |  |
| C (6 e-) | 17 | - | 17 | - | - |
| H (1 e-) | 35 | - | 35 | - | - |
|  |  |  |  |  |  |
| **Arachidonoyl tail** |  |  |  |  |  |
| C2-14 (6 e-) | 13 | - | 13 | - | - |
| H for C2-14 (1 e-) | 18 | - | 18 | - | - |
| C15-20 (6 e-) | 6 | - | - | 6 | 6 |
| H for C15-20 (1 e-) | 12 | - | - | 12 | 12 |
|  |  |  |  |  |  |
| **Oxidation** |  |  |  |  |  |
| O (8 e-) | - | - | - | 2 | - |
|  |  |  |  |  |  |
| **Total number electrons:** | **281** | **164** | **233** | **228** | **212** |
| **Calculated ρ/ρwater:** | **0.92** | **1.36** | **0.90** | **1.48** | **1.40** |


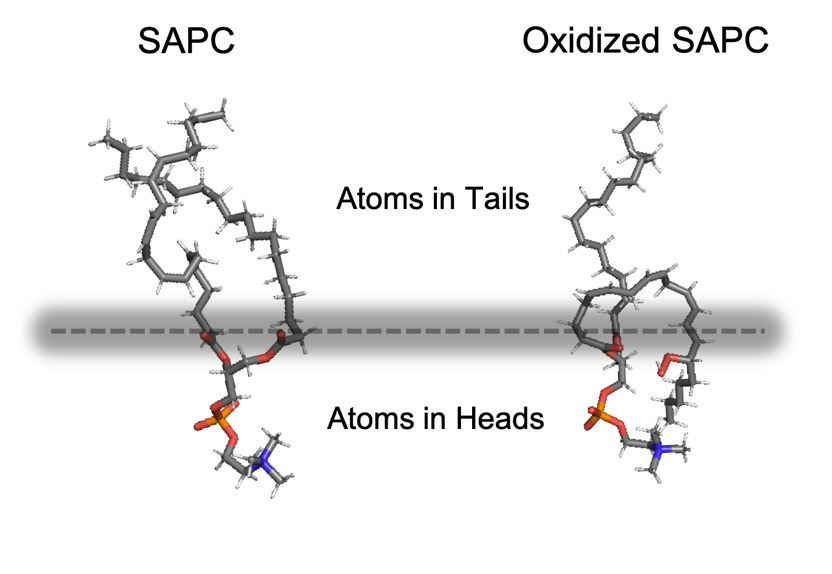

Supplement: supplement [file NIHMS2128675-supplement-supplement.docx]
